# Multi-Source Data-Driven Terrestrial Multi-Algorithm Fusion Path Planning Technology

**DOI:** 10.3390/s25123595

**Published:** 2025-06-07

**Authors:** Xiao Ji, Peng Liu, Meng Zhang, Chengchun Zhang, Shuang Yu, Bing Qi, Man Zhao

**Affiliations:** 1 China Satellite Network Digital Technology Co., Ltd., Xiong’an 070001, Chinaliup2@chinasatnet.com.cn (P.L.); zhangchch1@chinasatnet.com.cn (C.Z.); yush@chinasatnet.com.cn (S.Y.); qib@chinasatnet.com.cn (B.Q.); 2 School of Computer, China University of Geosciences (Wuhan), Wuhan 430074, China

**Keywords:** multi-source data, path planning algorithms, A* algorithm optimization, deep reinforcement learning, dynamic decision making

## Abstract

This paper presents a multi-source data-driven hybrid path planning framework that integrates global A* search with local Deep Q-Network (DQN) optimization to address complex terrestrial routing challenges. By fusing ASTER GDEM terrain data with OpenStreetMap (OSM) road networks, we construct a standardized geospatial database encompassing elevation, traffic, and road attributes. A dynamic-heuristic A* algorithm is proposed, incorporating traffic signals and congestion penalties, and is enhanced by a DQN-based local decision module to improve adaptability to dynamic environments. Experimental results on a realistic urban dataset demonstrate that the proposed method achieves superior performance in risk avoidance, travel time reduction, and dynamic obstacle handling compared to traditional models. This study contributes a unified architecture that enhances planning robustness and lays the foundation for real-time applications in emergency response and smart logistics.

## 1. Introduction

### 1.1. Research Background and Significance

With the continued development of urbanization, intelligent logistics, and emergency response systems, terrestrial transportation networks have become increasingly complex and dynamic. Efficient path planning is essential for improving transportation efficiency and ensuring safe, adaptive navigation across variable environments. Early research has explored foundational approaches including A*, Dijkstra, heuristic search, and optimization-based techniques [1,2,3,4], as well as comprehensive reviews on mobile robot navigation.

Recent studies have aimed to overcome limitations in static, single-objective planning by enhancing classical methods. For example, improved variants of the A* algorithm, such as chaos-based or hybridized models, have been shown to improve convergence in known-obstacle scenarios [5,6]. Deep reinforcement learning (DRL) approaches have further expanded the solution space, enabling agents to optimize trajectories under uncertainty [5,7,8,9,10,11,12,13,14,15,16].

Several domain-specific applications—including UAV patrol [17], agricultural drone routing [12], disaster rescue path estimation [18], and multi-missile or multi-agent tactical planning [14]—demonstrate the flexibility of DRL-enhanced planners across high-risk, multi-objective environments. In parallel, advancements in data-driven frameworks have emphasized the importance of integrating spatiotemporal data and multi-source fusion [19,20], including graph-based representations of urban road networks.

Beyond planning, robust localization under real-world constraints has also been explored. CNN-based outdoor positioning using 3D sensing [21] and deep learning models tailored for weather-varying conditions [22] underscore the need for perception-aware path optimization.

In contrast to most existing studies, which are primarily designed for autonomous platforms, our research focuses on path planning for human-driven urban vehicles. We address the practical need for dynamic route guidance that adapts to real-world constraints—such as congestion, signal delays, and restricted zones—while remaining computationally tractable and interpretable.

To this end, we propose a hybrid path planning framework that integrates multi-source heterogeneous data—such as satellite elevation, road topology, and real-time traffic—with a global–local algorithmic structure. Specifically, a dynamic heuristic A* module provides efficient global planning, while a Deep Q-Network (DQN) enables local adjustment based on context-aware priorities. This design supports intelligent, multi-objective route optimization in complex urban scenarios and contributes to the development of practical and deployable planning systems.

### 1.2. Research Objectives

Aimed at the path planning requirements for terrestrial target movement in spatiotemporal big data application scenarios, this study conducts comprehensive research on multi-source data-driven terrestrial multi-algorithm fusion path planning technology to satisfy diverse application demands. By integrating multi-source data (e.g., terrestrial elevation data and road network data) with multi-algorithm collaboration (Dijkstra, A*, ant colony optimization, and deep reinforcement learning), a dynamic adaptive path planning model is constructed to address the limitations of traditional single-source data perception in complex environments, delayed responses to dynamic obstacles, and conflicts in multi-objective optimization. The proposed technology enhances the global robustness, real-time performance, and multi-constraint coordination capabilities of path planning, thereby providing efficient and reliable comprehensive path decision-making support for terrestrial targets. To summarize, the main contributions of this study are as follows:

- We construct a standardized multi-source geospatial database by integrating ASTER GDEM terrain data with OpenStreetMap (OSM) road network information, enabling comprehensive terrain and traffic awareness.
- We propose a hybrid path planning framework that combines global A* search with local Deep Q-Network (DQN) optimization, achieving both global feasibility and local adaptability in dynamic environments.
- We introduce a dynamic multi-objective weighting mechanism tailored to diverse travel scenarios, balancing efficiency, safety, congestion, and route constraints in real time.
- We validate the effectiveness of the proposed method through extensive experiments on realistic urban road networks, demonstrating superior performance in risk control, route efficiency, and obstacle handling compared to traditional methods.

## 2. Preliminaries

**Figure 1:**
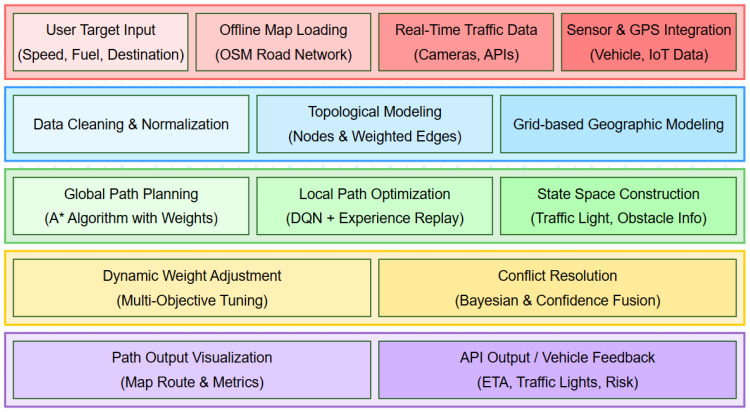
The refined system architecture of the proposed multi-source data-driven terrestrial path planning framework. It consists of five functional layers: (1) Data acquisition integration layer gathers user-defined targets and multi-source geographic data; (2) Preprocessing modeling layer constructs a unified topological and spatial representation; (3) Path planning core combines global A* and local DQN-based adjustment; (4) Adaptation conflict handling layer dynamically adjusts optimization priorities and resolves inconsistent inputs; (5) Output interaction layer provides real-time visualized results and vehicle feedback.

In addressing the optimization of urban path planning under constraints such as avoidance zones, congestion distance, and traffic signals, this study integrates the A* search algorithm with the Deep Q-Network (DQN) algorithm, as illustrated in the overall structure shown in Figure 1. The A* algorithm is employed to generate a global path, while the DQN algorithm performs local optimization. Furthermore, the heuristic function’s weight parameters are dynamically adjusted in response to varying travel scenarios. This section provides an overview of the A* algorithm, the DQN algorithm, and the dynamic weight adjustment mechanism. In addressing the optimization of urban path planning under constraints such as avoidance zones, congestion distance, and traffic signals, this study integrates the A* search algorithm with the Deep Q-Network (DQN) algorithm to form a hybrid decision-making framework that balances global optimality with local adaptability. Urban environments are dynamic and often involve complex conditions—such as sudden congestion, temporary road closures, and variable traffic light timings—that traditional single-layer path planning methods struggle to handle effectively.

In this framework, the A* algorithm is primarily responsible for generating an initial global path that satisfies static constraints and ensures overall efficiency from origin to destination. This path serves as the backbone for navigation, providing a high-level trajectory through the urban environment that avoids predefined restricted zones and minimizes the estimated travel distance.

On the other hand, the DQN algorithm acts as a local optimizer, refining the path in real time to adapt to dynamically changing traffic conditions. It is particularly effective in responding to unforeseen obstacles or localized disturbances, such as sudden traffic buildup or adaptive traffic signals, by making intelligent detours or re-routing decisions based on learned experience.

Moreover, to enhance the flexibility and contextual responsiveness of the planning system, the heuristic function within the A* algorithm is embedded with dynamically adjustable weight parameters. These parameters are fine-tuned during execution based on real-time travel scenarios—such as peak vs. off-peak hours or different urban regions—thereby allowing the system to prioritize different optimization goals (e.g., minimizing time vs. minimizing fuel consumption) as conditions evolve.

This section provides an overview of the collaborative use of the A* and DQN algorithms within the proposed hybrid framework, and introduces the adaptive weight adjustment mechanism that enables more intelligent and responsive urban path planning.

The terrestrial path planning task focuses on road network scenarios. By acquiring multi-modal geographic information and real-time dynamic data—including road network topology, traffic signal statuses, and road congestion indices—the heterogeneous data is processed through cleaning, format standardization, and spatial coordinate unification. This results in the construction of a standardized data repository for terrestrial transportation. Based on the processed data, road intersections are abstracted as network nodes, and road segment capacity is modeled as weighted edges. Leveraging the hierarchical characteristics of the road network, a topological network model is established. Through discretization modeling techniques, the continuous road network is transformed into a structured digital map compatible with path planning algorithms such as the A* algorithm and DQN algorithm. Ultimately, this methodology facilitates the development of an intelligent planning system incorporating real-time traffic situation awareness and dynamic route optimization.

The path planning module consists of the following key steps:

1. Input of Target Information
  The specific target information entered by the user via the frontend interface—such as speed, fuel consumption, and other relevant details—will be extracted for use in algorithmic calculations.
2. Data Processing
  Data processing operations involve the preloading of offline map data, data format conversion, and the integration of real-time traffic information.
3. Geographic Grid Construction
  Based on the processed data from the previous step, a geographic grid model is constructed, incorporating information such as avoidance points and restricted areas, to suit different algorithms.
4. Mode Selection
  For terrestrial path prediction, the user is required to select between online and offline modes.
5. Algorithm Calculation
  Based on the entered information and fundamental geographic data, an appropriate algorithm is selected to calculate the shortest path.
6. Solution Display
  The path planning results not only output the set of path points but also include information such as estimated travel time, estimated distance, the number of traffic lights, and congestion levels.

### 2.1. A* Algorithm for Path Planning

The A* algorithm (A-star algorithm) is a classical heuristic search algorithm that is widely applied in path planning and graph traversal problems. It has demonstrated outstanding performance in various domains, including robotic navigation, game AI, and mapping services. The core strength of the A* algorithm lies in its ability to guarantee the optimality of the solution while significantly enhancing search efficiency. Its fundamental principle involves initiating the search from a designated starting point and progressively exploring toward the goal, guided by an evaluation function that prioritizes paths with the highest potential to reach the destination—thereby avoiding exhaustive or uninformed searches.

**Figure 2:**
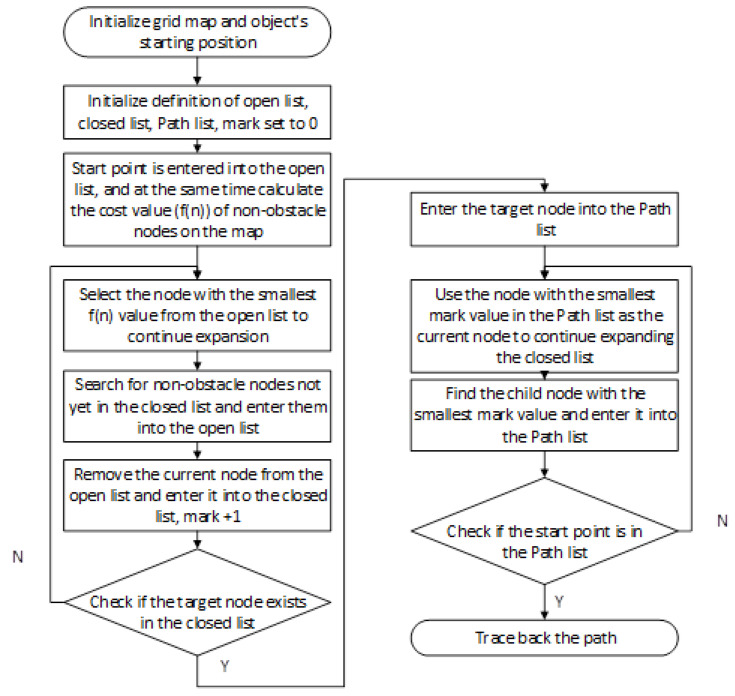
The basic flowchart of the A* algorithm.

At the core of the A* algorithm lies the evaluation function f(n), defined as f(n) = g(n) + h(n), where n denotes the current node under exploration. The term g(n) represents the actual cost incurred from the start node to node n, calculated as the cumulative cost along the known path. In contrast, h(n) is the estimated cost from node n to the goal node, commonly referred to as the heuristic function. The choice of heuristic function plays a critical role in determining both the efficiency of the algorithm and the quality of the resulting solution. Importantly, the heuristic does not need to provide an exact prediction of the remaining cost—it suffices that the estimate does not exceed the true cost (a property known as admissibility). When this condition is met, the A* algorithm is guaranteed to produce an optimal path.The basic structure of this algorithm is illustrated in Figure 2.

During execution, the algorithm maintains two sets: the open list and the closed list. The open list contains nodes that are pending exploration, while the closed list records nodes that have already been processed. At each iteration, the algorithm selects the node with the smallest f(n) value from the open list as the current node for expansion. It then evaluates all the neighboring nodes of the current node. For each neighbor, the algorithm computes updated values for g, h, and f, and based on these values, either adds the neighbor to the open list or updates its information if a more efficient path is found. The current node is subsequently added to the closed list to prevent redundant processing.

### 2.2. Deep Q-Network Algorithm

The Deep Q-Network (DQN) is an optimization algorithm that integrates deep learning with reinforcement learning, designed to address decision-making problems in high-dimensional state spaces. Its core concept lies in leveraging deep neural networks to approximate the Q-function, enabling the agent to learn the state–action value function Q(s,a) and thereby derive optimal decision policies within its environment.

In conventional Q-learning, the storage and iterative update of a Q-value table are feasible only when the state space is relatively small. However, this approach becomes impractical in high-dimensional or continuous state spaces. DQN addresses this limitation by employing a deep neural network as a function approximator, replacing the Q-table with a parameterized network. This innovation significantly enhances the applicability of reinforcement learning in complex task domains.

The primary procedure of DQN is as follows: at each time step *t*, the agent receives the current state $s_{t}$ from the environment and selects an action based on an ε-greedy policy—that is, with probability ε, it selects a random action, and with probability 1−ε, it selects the action with the highest estimated Q-value. Upon executing the action, the environment returns the next state $s_{t+1}$ and an immediate reward $r_{t}$.

**Figure 3:**
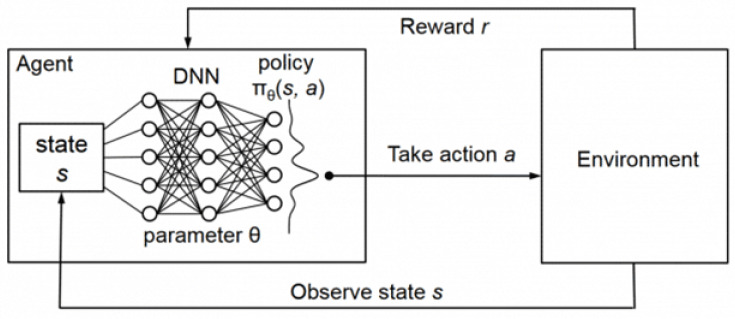
Framework diagram of the DQN algorithm.

A critical component of DQN is the experience replay mechanism. The agent stores each interaction tuple $\left(s_{t},a_{t},r_{t},s_{t+1}\right)$ in a replay buffer and samples mini-batches randomly during training. This breaks the temporal correlations inherent in sequential data, thereby enhancing the stability and efficiency of the learning process, as illustrated in Figure 3.

To further stabilize training, DQN introduces the target network mechanism. Specifically, it maintains two networks: a primary network Q(s,a;θ), which is updated continuously, and a target network $Q^{\prime }\left(s,a;\theta^{-}\right)$, which periodically synchronizes its parameters with the primary network. The target Q-value is updated using the following formulation:

(1) $$y_{t}=r_{t}+\gamma \max_{a^{\prime }}Q^{\prime }\left(s_{t+1},a^{\prime };\theta^{-}\right)$$

Here, γ denotes the discount factor, representing the importance of future rewards. The network parameters θ are optimized by minimizing the mean squared error loss function:

(2) $$L\left(\theta \right)=E_{(s,a,r,s^{\prime })∼D}\left[{\left(y_{t}-Q\left(s,a;\theta \right)\right)}^{2}\right]$$

Through iterative updates, DQN refines the network parameters θ to approximate the optimal Q-function, thereby improving and reinforcing the policy.

By integrating mechanisms such as deep neural networks, experience replay, and target networks, DQN demonstrates robust learning capabilities in high-dimensional environments and has been successfully applied to complex tasks such as robotic path planning.

### 2.3. Dynamic Weight Design

To better accommodate the increasingly complex demands of urban and intercity transportation, we propose a multi-objective dynamic weight adjustment mechanism grounded in real-world traffic scenarios. This mechanism dynamically modifies the weight distribution among multiple objective functions in the route planning process, based on environmental perception and scenario recognition outcomes. In doing so, it enables precise responsiveness to user requirements and adaptive optimization across diverse travel contexts.

During the weight adjustment process, the system accounts for potential correlations among various objectives. For instance, total travel time, total distance, and expressway usage often exhibit positive correlation. Improper weight allocation in such cases may result in the overdominance of a single objective, thereby undermining the overall fairness of the optimization outcome. To address this, we introduce a correlation suppression module that automatically reduces the joint influence of strongly correlated objectives, promoting a more balanced optimization result. Furthermore, the newly introduced avoidance zone feature enhances the system’s ability to circumvent high-risk or sensitive areas, thereby improving safety.The weight configurations corresponding to different travel scenarios are summarized in Table 1.

In light of increasingly diverse urban travel demands, differentiated weight configurations for route planning are designed for specific scenarios to align better with real-world usage requirements:

Daily commutes prioritize travel efficiency and smooth traffic flow, emphasizing time minimization and the avoidance of congested routes to ensure a stable and efficient commuting experience.

Long-distance travel places greater emphasis on overall route economy and safety, focusing on path optimization and risk avoidance to ensure a smooth and secure journey.

School pickup and drop-off prioritizes safety above all, with particular attention to avoiding complex road segments and potential hazard zones, thereby ensuring a secure and manageable environment for child transportation.

Night-time deliveries emphasize driving safety and risk mitigation under low-light conditions, while also considering delivery efficiency to maintain operational stability and reliability during off-peak hours.

### 2.4. Conflict Resolution in Multi-Source Data Fusion

In scenarios where information from different sources conflicts—e.g., GPS data indicates a free road while traffic cameras detect congestion—effective conflict resolution mechanisms become essential. Our current model prioritizes GPS- and OSM-based traffic data, but we acknowledge the need for integrating real-time video surveillance or transport authority reports to improve trustworthiness. In future work, we will explore data reliability scoring models and fusion consistency checks to enhance the robustness of multi-source decisions. Potential techniques include confidence-weighted fusion, historical consistency verification, and Bayesian inference to better evaluate and reconcile conflicting inputs.

## 3. Multi-Objective Path Planning

In the study of multi-objective route planning, this research focuses on five key objectives for in-depth analysis and optimization, aiming to enhance the driving experience across various application scenarios and to improve the efficiency and quality of route planning.

### 3.1. Optimization Objectives

In this study, the weighted sum method (WSM) is adopted as the core multi-criteria decision-making (MCDM) approach for multi-objective route planning. WSM offers a clear structure and ease of implementation, along with strong robustness to fluctuations in weight assignments, making it well-suited for the demands of dynamically changing traffic scenarios. In contrast, other mainstream MCDM methods, such as the multiplicative aggregation method, may introduce distortions due to nonlinear interactions among objectives. Similarly, while the technique for order preference by similarity to an ideal solution (TOPSIS) performs well in ranking alternatives, it suffers from high computational complexity and normalization bias in high-dimensional dynamic path optimization problems. Considering algorithmic efficiency, controllability, and adaptability to real-world applications, WSM remains the most suitable choice for this research.

The multi-objective route planning problem formulated in this study seeks to minimize a composite optimization function $F_{\mathrm{total}}\left(p\right)$, which consists of a set of weighted objective functions. A route *p* is defined as a sequential path of nodes $p=(n_{1},n_{2},\ldots ,n_{k})$ from the origin to the destination, where each pair of adjacent nodes $\left(n_{i},n_{i+1}\right)$ is connected by an edge in the graph *G*. The objective function is composed of multiple weight factors and normalized objective metrics. Each weight dynamically varies according to the travel scenario to reflect the diversity and adaptability of user requirements. To ensure comparability among different objectives, all terms are normalized using a common formula. The final mathematical formulation of the optimization objective is expressed as follows:

(3) $$\begin{array}{cc} \min_{p}\;F_{\mathrm{total}}\left(p\right)=\; & \;\alpha \left(t\right)\cdot f_{\mathrm{length}}\left(p\right)+\beta \left(t\right)\cdot f_{\mathrm{congestion}}\left(p\right)+\gamma \left(t\right)\cdot f_{\mathrm{time}}\left(p\right)\\ \; & +\epsilon \left(t\right)\cdot f_{\mathrm{risk}}\left(p\right)+\eta \left(t\right)\cdot f_{\mathrm{avoidance}}\left(p\right) \end{array}$$

As summarized in Table 2, the objective functions and their descriptions are as follows:

To ensure the feasibility and safety of the generated routes, the following formulation defines the set of constraints for the optimization problem:

(4) $$\begin{array}{cc} \; & f_{\mathrm{time}}\left(p\right)\leq T_{\max}\\ \; & f_{\mathrm{congestion}}\left(p\right)\leq C_{\max}\\ \; & f_{\mathrm{risk}}\left(p\right)\leq R_{\max}\\ \; & f_{\mathrm{avoidzone}}\left(p\right)\leq A_{\max} \end{array}$$

Here, $T_{\max},\;C_{\max},\;R_{\max},\;A_{\max}$ denote the maximum tolerable thresholds for travel time, congestion, risk, and avoidance zone exposure, respectively. These thresholds are imposed to prevent the selected route from deviating beyond the bounds of safety and feasibility. In addition, the constraints on the decision variables within the optimization problem are defined as follows:

(5) $$\forall i\in \left\{1,2,\ldots ,k-1\right\},\;\left(n_{i},n_{i+1}\right)\in E$$

That is, each pair of adjacent nodes in the route must be connected by a valid edge in the graph *G* to ensure the physical accessibility of the path within the actual road network.

This study selects five key optimization objectives: total distance, congested distance, travel time, risk index, and avoidance zone. These objectives collectively reflect the multi-dimensional performance of travel routes in terms of efficiency, safety, and driving preferences. They enable dynamic weight adjustment and personalized route planning based on the actual needs of the user and the environment. The definitions and calculation methods for each optimization objective are as follows:

1. **Path Length**

To determine the optimal vehicle route from origin to destination, the total distance of the path serves as a fundamental optimization metric. Given a sequence of path nodes $p=\{n_{1},n_{2},\ldots ,n_{k}\}$, the total path length $f_{\mathrm{length}}\left(p\right)$ is computed as

(6) $$f_{\mathrm{length}}\left(p\right)=\sum_{i=1}^{k-1}d\left(n_{i},n_{i+1}\right)$$

where $d(n_{i},n_{i+1})$ denotes the Euclidean or actual road distance between consecutive nodes. This metric forms a core component of the overall cost function used to evaluate and optimize paths.

After determining a feasible sequence of waypoints, a genetic algorithm is applied to perform global optimization, aiming to minimize path length while also considering additional criteria such as smoothness and safety. The process involves the following steps:

- **Population Initialization**: Randomly generate an initial population of *N* candidate paths, each representing a full traversal through the predefined waypoints.
- **Fitness Evaluation:** Evaluate each path using a composite cost function that integrates the total path length $f_{\mathrm{length}}\left(p\right)$, path smoothness, and proximity to obstacles (safety margin).
- **Selection operation**: Retain the top 20% of elite individuals based on fitness scores, and select the remaining 80% via roulette wheel selection to maintain genetic diversity.
- **Crossover and Mutation**:
  - *Two-point crossover*: Exchange middle segments between pairs of parent paths to create offspring.
  - *Gaussian mutation*: Apply perturbations to waypoint coordinates with a probability of 5%, promoting the exploration of the solution space.
- **Iterative Convergence**: Repeat the evaluation, selection, and variation steps for up to 100 generations or until the fitness standard deviation across the population drops below 0.1%.
- **Optimal Solution Output**: The individual with the highest fitness in the final generation is selected as the globally optimized path.

By integrating path length as a primary component of the fitness function and employing evolutionary strategies, this approach effectively searches for routes that are not only short, but also smooth and safe for vehicle traversal.

2. **Congestion Length**

Congested distance is used to measure the length of congested segments along the route, reflecting the traffic flow pressure. The congestion state of each road segment is determined by its congestion label, and the calculation formula is as follows:

(7) $$f_{\mathrm{congestion}}\left(p\right)=\sum_{i=1}^{k-1}\;\left\{\begin{array}{cc} d(n_{i},n_{i+1}) & \mathrm{if}\;\mathrm{congestion}(n_{i},n_{i+1})>0\\ 0 & \mathrm{otherwise} \end{array}\right.$$

Here, $\mathrm{congestion}(n_{i},n_{i+1})$ represents the congestion label of the edge. A non-zero value indicates that the segment is congested.

3. **Travel Time**

Total travel time refers to the cumulative time required for the vehicle to complete the entire route, taking into account the traffic speed limits and real-time traffic conditions. Let $t(n_{i},n_{i+1})$ represent the estimated travel time between nodes $n_{i}$ and $n_{i+1}$, then

(8) $$f_{\mathrm{time}}\left(p\right)=\sum_{i=1}^{k-1}\;t\left(n_{i},n_{i+1}\right)$$

**Figure 4:**
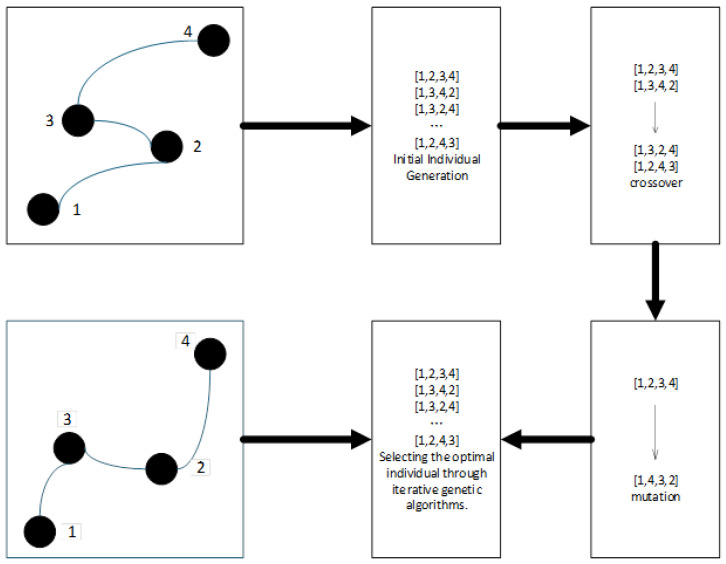
The path planning of waypoints without sequence.

This metric is particularly critical for peak-hour scheduling and emergency response route planning, and the process of unordered waypoint optimization is illustrated in Figure 4.

4. **Risk Index**

The risk index is used to measure the potential safety risks along the route, considering factors such as historical accidents, night-time lighting, weather conditions, and public security. The total risk index for the route is calculated as follows:

(9) $$f_{\mathrm{risk}}\left(p\right)=\sum_{i=1}^{k-1}\;r\left(n_{i},n_{i+1}\right)$$

Here, $r(n_{i},n_{i+1})$ represents the risk factor value of the edge. A higher value indicates greater safety risk.

5. **Avoidance Zone**

The avoidance zone index reflects the extent to which the route traverses specific sensitive areas, such as construction zones, accident-prone areas, and restricted zones. Let the area avoidance factor be $a(n_{i},n_{i+1})$, where a value of 1 indicates that the edge is within an avoidance zone. The calculation of the avoidance zone index is as follows:

(10) $$f_{\mathrm{avoidzone}}\left(p\right)=\sum_{i=1}^{k-1}\;\left\{\begin{array}{cc} d(n_{i},n_{i+1}) & \mathrm{if}\;a(n_{i},n_{i+1})=1\\ 0 & \mathrm{otherwise} \end{array}\right.$$

The objective is to minimize this value in order to enhance the safety and traffic efficiency of the route.

The aforementioned optimization objectives are designed to comprehensively assess the quality of a route from multiple key dimensions, in order to meet the diverse needs of vehicle route planning within intelligent transportation systems. In different application scenarios, the importance of each optimization objective may vary. By introducing a dynamic weight adjustment mechanism, the system is able to flexibly adjust the relative importance of each objective according to the specific requirements of a given scenario, thereby providing more optimized route selections.

### 3.2. Optimization Method

This experiment utilizes Deep Q-Learning (DQN) in conjunction with the A* algorithm for multi-objective path optimization. By combining the autonomous decision-making ability of the agent with the advantages of heuristic search, a dual-path generation mechanism is established to explore the multi-objective optimization space. The A* algorithm ensures the basic feasibility of the solution, while the DQN network learns dynamic optimization strategies through environmental interactions. Together, they generate a set of paths that satisfy multi-objective constraints.

The method integrates the advantages of both path generation approaches through the experience replay mechanism: A* paths serve as high-quality samples to accelerate DQN convergence, while the DQN-explored paths iteratively optimize the Q-value function through the Bellman equation, dynamically adjusting the weights of the path evaluation metrics. For each generated path, a multi-objective value network is employed to simultaneously assess various path attributes (such as distance, risk factor, etc.), and the advantage function decomposition technique is applied to achieve trade-off optimization across multiple objectives. This hybrid path generation strategy ensures the feasibility of the solution set, while leveraging the generalization ability of deep neural networks to explore a broader solution space.

In the first phase, the A* algorithm is employed for global path search. The core of this approach lies in the introduction of a multi-objective dynamic weighted cost function, which comprehensively evaluates five optimization objectives along the path: total distance, congested distance, travel time, risk index, and coverage of avoidance areas. The A* algorithm calculates the priority of each node using the heuristic evaluation function (f (n) = g (n) + h (n)). During path expansion, the algorithm dynamically determines whether an edge belongs to an avoidance area or restricted road segment based on its attributes, and adjusts the weights of each optimization objective according to the current travel scenario. This adjustment reduces the bias in overall path selection caused by the high correlation between objectives. Ultimately, the A* algorithm generates a globally feasible path that satisfies multi-objective constraints (such as minimizing congestion, travel time, or risk), providing a framework for subsequent local optimization.

After obtaining the initial path from the A* algorithm, the second phase introduces the deep Q-network (DQN) model from deep reinforcement learning to perform local path optimization. In this phase, the A* path serves as the input, and the state space is constructed by incorporating current traffic conditions, road status, traffic light information, reachable neighboring nodes, and obstacle avoidance requirements, which serve as environmental perception data. The Q-value function is then used to assess the expected return of the current node under different actions (i.e., moving to the next neighboring node), thus enabling dynamic optimization of the selection of local nodes, see Algorithm 1.

   **Algorithm 1:** Dynamic-weighted A* with DQN for multi-objective path optimization.      **Inputs:** *G*: Road network graph, $n_{\mathrm{start}},n_{\mathrm{goal}}$: Start and goal nodes      (waypoints, avoid_points, avoid_zones): Pathway points and avoidance areas      (traffic_lights, road_conditions): Traffic light status and road conditions      $T_{\max},C_{\max},R_{\max},A_{\max}$:    Constraint thresholds for time, congestion, risk,      and avoidance areas      α(t),β(t),γ(t),ϵ(t),η(t):    Dynamic weight factors for different objectives at time *t*      **Output:** optimized_path: The optimized path from $n_{\mathrm{start}}$ to $n_{\mathrm{goal}}$      1   Initialize openSet, closeSet      2   Add $n_{\mathrm{start}}$ to openSet with priority 0      3   **while** openSet is not empty do      4      n← node in openSet with lowest $F_{\mathrm{total}}\left(n\right)$      5      **if** $n==n_{\mathrm{goal}}$      6         global_path← trace back from $n_{\mathrm{goal}}$ to $n_{\mathrm{start}}$      7         **break**      8      **end if**      9      Remove *n* from openSet; add to closeSet      10      **for each** neighbor *m* of *n*      11         **if** *m* in closeSet or *m* in avoid_points or *m* in avoid_zones      12            **continue**      13         **end if**      14         Compute dynamic weights α(t),β(t),γ(t),ϵ(t),η(t)      15         Compute multi-objective cost $F_{\mathrm{total}}\left(m\right)$:                        $F_{\mathrm{total}}\left(m\right)=\alpha \cdot f_{\mathrm{length}}+\beta \cdot f_{\mathrm{congestion}}+\gamma \cdot f_{\mathrm{time}}+$                        $\eta \cdot f_{\mathrm{risk}}+\epsilon \cdot f_{\mathrm{avoidzone}}$      16         Set parent of m←n      17         Add *m* to openSet      18      **end for**      19   **end while**

The agent employs an ϵ-greedy strategy to balance exploration and exploitation, continuously sampling training samples from the experience replay buffer to iteratively update the Q-network. This process enhances the ability of the path to respond to unexpected situations. The DQN model not only improves the adaptability and safety of the path at the local level, but also allows the path to avoid potential risk areas at specific nodes, enabling the fine-tuning of the path to adapt to real-time environmental changes.

The combination of A* and DQN in the path planning task achieves a collaborative effect of “global optimality + local adaptation”. The A* algorithm ensures the multi-objective feasibility and efficiency of the path in terms of overall structure, making it suitable for global optimal path search. On the other hand, DQN leverages the advantages of reinforcement learning in dynamic environment modeling and decision optimization, compensating for A*’s slower response to environmental changes. The synergy between the two enhances the path planning system by providing strong objective control while maintaining the flexibility to adapt to dynamic and complex traffic environments, thus improving the system’s intelligence, practicality, and robustness, see Algorithm 2.

   **Algorithm 2:** Local path optimization using DQN.      **Inputs:** global_path: The global path to be optimized      (traffic_lights,road_conditions): Traffic light status and road conditions      (avoid_points,avoid_zones): Points and areas to avoid during path optimization      ϵ: Exploration factor for the DQN policy      α,β,…,θ: Weights for multi-objective reward computation      **Output:** optimized_path: The optimized path from start to goal after local adjustments      1   Initialize Q-network and experience replay buffer      2   optimized_path ← [global_path[0]]      3   current_node ← global_path[0]      4   **for** i=1 **to** len(global_path) **do**      5      current_state←get_state(current_node,traffic_lights,road_conditions,                 avoid_points,avoid_zones)      6      **if** random() < ϵ **then**      7         action ← random neighbor of current_node      8      **else**      9         action ← select_best_action(current_state)      10      **end if**      11      next_node ← action      12      **if** next_node ≠ global_path[i] **then**      13         optimized_path.append(next_node)      14      **end if**      15      next_state←get_state(next_node,traffic_lights,road_conditions,                 avoid_points,avoid_zones)      16      reward ← compute_reward(next_node, α,β,…,θ)      17      store_transition(current_state, action, reward, next_state)      18      **if** replay_buffer is full **then**      19         batch ← sample_batch()      20         **for each** transition in batch      21            update_Q_network(transition)      22         **end for**      23      **end if**      24      current_node ← next_node      25   **end for**      26   **return** optimized_path

## 4. Experimental Design

This experiment is designed to evaluate the path planning performance of a hybrid strategy that combines global path planning using the A* algorithm with local path optimization via deep Q-networks (DQN), in both static and dynamic environments. The proposed approach is compared against traditional single-algorithm methods in terms of path length, computational efficiency, and obstacle avoidance capability.

### 4.1. Experimental Data and Environment

The road network data used in this experiment is derived from the offline OpenStreetMap (OSM) dataset for the Wuhan metropolitan area, downloaded in December 2024. After preprocessing, the dataset retains key road categories—such as motorway, primary, and residential—as well as traffic light information. These data are structured into a graph format to support path computation. The experimental route spans from Wuhan Tianhe International Airport to Wuhan Railway Station, covering a variety of road types including expressways, urban arterial roads, secondary roads, and residential streets. This route exhibits a high degree of structural complexity and serves as a representative case for evaluating path planning performance. The planning process relies solely on static traffic information and does not incorporate real-time traffic updates. The reinforcement learning component is trained and evaluated within a simulated environment constructed based on the OSM-derived map. The state space consists of local map features, the agent’s current position, and the target location; the action space includes directional movements and detour maneuvers.

To emulate multi-source data fusion in a cost-effective and reproducible way, this study utilizes third-party APIs that provide synthetic representations of real-world sensor streams. These include the following:

- **GPS-derived trajectory data**: Emulates real-time vehicle positioning and routing.
- **Traffic congestion APIs**: Simulate external sensing from road-side cameras or inductive coils by providing region-specific congestion scores and delay estimates.
- **Environmental status inputs**: Include information such as blocked roads or traffic incident alerts, analogous to what might be obtained from radar or IoT-based city infrastructure.

These sources are integrated into the planning model through feature normalization and timestamp alignment, forming the input tensor for our fusion-based trajectory planning framework. While the physical sensor implementation is beyond our current experimental scope, our framework remains extensible to real-world deployment with minimal structural changes.

### 4.2. Hybrid Algorithm Strategy Workflow

The OpenStreetMap (OSM) data is modeled as a directed graph G=(V,E), where nodes *V* represent intersections or traffic signal-controlled junctions, and edges *E* denote road segments between these nodes. Each edge is annotated with multi-dimensional attributes including

- **Static features:** path length, road classification (e.g., highway, residential), bidirectional accessibility.
- **Dynamic features:** real-time congestion levels, traversal cost penalties (e.g., 60 s delay for traffic light waiting).
- **Operational constraints:** predefined avoidance zones or prohibited areas.

Nodes *V* are enriched with contextual attributes such as traffic signal presence, restricted zone status, and waypoint markers. During the global planning phase, critical spatial constraints (e.g., avoidance points, restricted zones) are integrated into the A* algorithm’s heuristic function to guide path exploration. The A* algorithm generates an initial optimal path by minimizing a composite cost function that balances distance, traffic penalties, and constraint violations.

For dynamic adaptation, temporary obstacles (e.g., simulated traffic incidents) are injected into the navigation process. The road network graph is continuously updated with real-time edge attributes (e.g., congestion levels, temporary closures) and node states (e.g., signal timing changes). The Deep Q-Network (DQN) agent then performs local path refinement through reinforcement learning, leveraging its state space representation of adjacent nodes, edge weights, and obstacle distributions to optimize detour decisions while maintaining alignment with the global A*-derived trajectory.To validate the effectiveness of different strategies, we design three comparative experiments based on varying levels of global and local integration, as summarized in Table 3.

This hybrid architecture enables synergistic coordination between deterministic global optimization and data-driven local adaptation within the structured graph representation.

**Figure 5:**
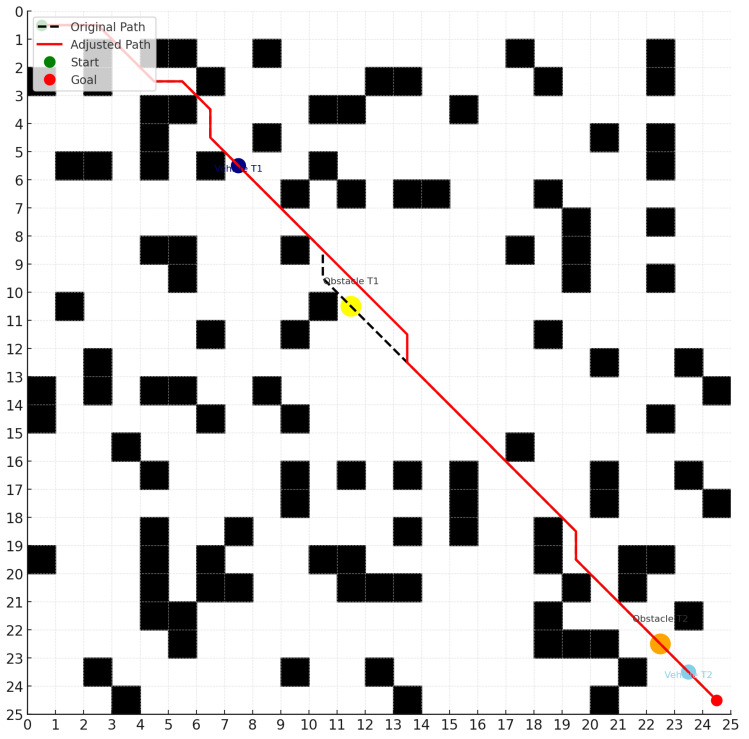
Comparison of planned paths with and without dynamic obstacles. The schematic illustrates the vehicle’s path adjustment in response to a moving obstacle, shown at two time points (T1 and T2). The dark blue circle indicates the vehicle’s position at T1, while the yellow circle represents the obstacle’s position at T1. The light blue circle marks the vehicle’s position at T2, and the orange circle denotes the obstacle’s position at T2.

The specific experimental procedure is as follows:

1. Initialize the map, including the start point, end point, and obstacle distribution.
2. Plan a global path using the A* algorithm.
3. Introduce dynamic obstacle regions during the simulation process.
4. As the agent traverses the path, it performs local optimization, obstacle avoidance, and rerouting through the DQN module, as illustrated in Figure 5.
5. Repeat the experiment 100 times and record the experimental data.
6. Conduct statistical analysis to compare the performance of each algorithm in terms of path length, planning time, and risk coefficient.

### 4.3. Limitations Analysis

Although this experiment was designed to closely approximate real-world scenarios, several limitations remain, as detailed below: 1. OSM data inaccuracies: The road network data derived from OpenStreetMap (OSM) may contain errors such as the incorrect classification of road hierarchies or missing annotations of traffic signals, which can undermine the reliability of path planning outcomes. 2. Idealized assumption of positioning information: The experimental setup employs a simulated environment for path planning tests, assuming perfectly accurate positioning information. It does not account for real-world phenomena such as GPS signal drift, occlusion, or measurement errors. Consequently, the deep Q-network (DQN) module’s training and decision-making are based on an ideal state estimate, and its robustness under perceptual uncertainty has not been evaluated. 3. Lack of dynamic traffic modeling: The road data used are offline OSM maps, where path costs consider only static attributes such as road types and traffic signal locations. Realistic traffic dynamics—including variable traffic flow, incidents, or road closures—are not simulated, and thus the effectiveness of the A* algorithm’s planned routes under highly dynamic conditions remains unverified. 4. Static traffic signal states: The experiment assumes fixed-traffic signal layouts and timing plans, without accounting for adaptive signal control strategies that vary signal phases in response to traffic conditions. This simplification limits the temporal accuracy of path cost assessments related to traffic signal delays.

## 5. Discussion

### 5.1. Result Analysis

This experiment was conducted within a simulated environment and did not account for factors such as localization errors, perception uncertainties, or dynamic traffic variations that may occur in real-world driving conditions. The specific experimental results are presented in Table 4, comparing the performance of A*, DQN, and the proposed A*–DQN hybrid method across four evaluation metrics.

In quantitative terms, the proposed hybrid approach achieves a 4.1% increase in path length over the theoretical optimum (A*), while reducing the risk coefficient by 55.6% compared to A*, and improving the obstacle avoidance success rate from 10% to 95%. Planning time is also reduced by 61.9% relative to DQN-only methods.

The analysis of data from 100 repeated experiments revealed significant differences among the three algorithms across multiple performance metrics. In terms of path length, the A* algorithm produced a 31.5 km route under static conditions, reflecting theoretical optimality. However, its inability to adapt to dynamic obstacles severely limits its practical applicability. The pure DQN approach, lacking global path guidance, generated redundant detours during obstacle avoidance, resulting in an average path length of 38.2 km—an increase of 21.6% compared to the optimal solution. In contrast, the hybrid A-DQN algorithm demonstrated a well-balanced performance. It achieved an average path length of 32.8 km, only 4.1% longer than the theoretical optimum. This difference was confirmed to be statistically significant through a paired-sample t-test (*p* < 0.01). These findings validate the dual advantages of the hybrid algorithm: maintaining the global path optimization capability of A* while effectively handling dynamic obstacles through local DQN-based adjustments.

The analysis of the planning time metric reveals distinct computational efficiency characteristics among the algorithms. The A* algorithm, leveraging its deterministic search strategy, completed path planning in static environments within just 0.3 s. In contrast, the pure DQN approach, which relies on real-time neural network inference, exhibited an average planning time of 2.1 s—a potential bottleneck in applications with stringent real-time requirements. The A-DQN hybrid algorithm incorporates an intelligent triggering mechanism that activates the DQN module only upon detecting a potential collision risk. As a result, the overall planning time is maintained under 0.8 s, representing a 61.9% improvement in efficiency compared to the pure DQN method. This approach effectively balances computational complexity and real-time performance demands.

**Figure 6:**
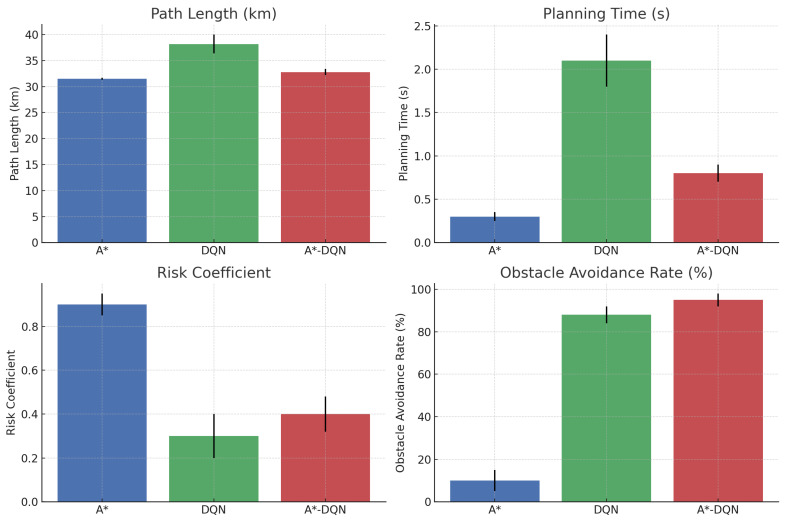
Comparison of path length, planning time, risk coefficient, and obstacle avoidance rate for A*, DQN, and A*-DQN.

In terms of safety assessment, this study proposes a risk coefficient model that comprehensively accounts for both collision risk and path deviation. The analysis reveals that the A* algorithm, which entirely disregards dynamic obstacles, yields a high risk coefficient of 0.9, indicating significant safety concerns in real-world applications, as shown in Figure 6. The pure DQN method demonstrates a more conservative approach, reducing the risk coefficient to 0.3. However, its excessive avoidance behavior negatively impacts path smoothness. The A-DQN hybrid algorithm achieves a risk coefficient of 0.4, effectively balancing safety with path continuity. Further evidence is provided by the dynamic obstacle avoidance success rate: the hybrid algorithm achieves a 95% success rate, substantially outperforming the A* algorithm (10%) and exceeding the pure DQN method (88%). This improvement is particularly notable in complex scenarios such as sudden lane changes by surrounding vehicles, where the hybrid approach demonstrates superior decision-making in obstacle avoidance.

### 5.2. Discussion of Method Effectiveness

The ground-based multi-algorithm fusion path planning technique driven by multi-source data proposed in this study effectively makes up for the lack of real-time adaptability and the global optimization capability of traditional path planning methods in complex dynamic environments. Previous studies have mostly focused on a single algorithm (e.g., A*, Dijkstra, or reinforcement learning models) or relied on limited data sources (e.g., static maps or GPS trajectories), which show limitations when dealing with scenarios such as unstructured road conditions, variable weather, and traffic bottlenecks.

The contributions of this study are reflected in the following aspects:

1. **Multi-source heterogeneous data fusion mechanism:** Unlike traditional path planning that relies only on GPS or static map information, this study organically integrates satellite navigation, ground sensing (e.g., cameras, inductive coils), and real-time traffic flow data, which provides a richer and more dynamic environment sensing support for path decision making.
2. **Innovative multi-algorithm fusion architecture:** By combining the heuristic global search capability of the A* algorithm and the policy self-learning advantage of DQN in complex local scenes, better path planning performance is realized. Compared with the existing literature that applies A* and deep reinforcement learning separately, this study demonstrates the performance improvement after their fusion: in the simulation test of an urban road network, the average time consumed for planning paths is reduced by 61.90% compared with DQN; the risk factor is reduced by 55.56% compared with A*; and the success rate of dynamic obstacle avoidance is improved by 8.5 times.
3. **Improvement of system robustness and adaptability:** The method can be dynamically adapted to heterogeneous terrains, such as dense urban road sections, rugged roads in mountainous areas, and transportation hubs, which significantly improves the environmental adaptability of path planning. Compared with the DQN-only approach, the fusion strategy shows higher stability and response efficiency in dynamic obstacle prediction and unexpected event response.
4. **Complementary to existing research:** Most of the current literature focuses on the improvement of the algorithm performance itself, and less on the algorithm synergy mechanism supported by multi-source data. This study fills the gap and systematically proposes a complete path optimization framework from data fusion, algorithm design to system integration, which has strong engineering promotion potential and academic innovation value.

## 6. Conclusions

This study presents a multi-objective path planning method tailored for typical urban traffic scenarios. The framework integrates A*search for global path computation with a Deep Q-Network (DQN) module for local refinement, enabling coordinated planning that balances efficiency, safety, and adaptability.

To support context-aware optimization, a dynamic weight adjustment mechanism is introduced, allowing real-time prioritization among competing objectives such as travel time, congestion, risk, and avoidance zones. Multi-source data—including real-time traffic, historical usage patterns, and sensor-derived information from GPS and traffic cameras—are fused to enhance environmental awareness and decision quality. These trade-offs are particularly evident under varying travel scenarios. For example, A* performs well in predictable environments with static conditions, such as low-traffic or structured routes. In contrast, DQN excels in dynamic or uncertain settings, where obstacle avoidance and risk mitigation are critical. The hybrid approach adapts across these contexts, leveraging scenario-dependent objective weights.

This balance reflects the classic exploration–exploitation trade-off in planning under uncertainty. A* emphasizes the deterministic exploitation of known paths, while DQN introduces learning-based exploration to handle unmodeled disruptions. Their integration enables both robust guidance and adaptive responsiveness.

Experimental evaluations based on OpenStreetMap road network data demonstrate that the proposed method achieves superior performance across multiple real-world scenarios. Compared with Global A* and DQN-only approaches, the hybrid A*-DQN framework exhibits shorter paths than DQN, and lower risk than A*, validating the advantage of combining global search efficiency with local adaptability. Moreover, the results highlight the importance of dynamic weighting, which enables the planner to adapt its behavior to varying travel contexts, such as rush hour or night-time delivery.

## 7. Future Work

Although recent advances in path planning have demonstrated promising results, many current studies still face two critical limitations. First, most rely on single-source data—such as static maps or GPS traces—which fail to capture the complexity of dynamic urban environments involving real-time traffic, terrain variability, and unexpected disturbances. Second, many approaches employ a single algorithmic strategy (e.g., A*, Dijkstra, or reinforcement learning) in isolation, lacking the flexibility to balance global optimality with local adaptability. These limitations reduce the robustness and real-time responsiveness of path planning systems.

Our current framework addresses these issues through a hybrid design that integrates multi-source data and combines global A* with local DQN-based optimization. However, further improvements are still needed. Specifically, our study is currently limited to single-agent settings, simplified obstacle modeling, and simulation-based environments without real-world integration. Future work will explore the following directions:

- **Multi-agent interactions:** Extend the framework to handle cooperative or competitive scenarios among multiple vehicles.
- **Dynamic obstacle behavior:** Incorporate realistic and probabilistic models for moving entities such as pedestrians and other vehicles.
- **Scalability in large-scale urban networks:** Conduct large-scale tests using city-wide maps with real-time traffic data.
- **Integration with control and localization:** Combine the planning module with vehicle dynamics, MPC control, and GPS/HD-map-based localization for real-world deployment.

## Figures and Tables

**Table 1:** Weighting of transport demand in multi-scenario cities.

Travel Scenario	Time	Congested Distance	Total Distance	Safety	Avoidance Zone
Daily Commute	0.30	0.30	0.25	0.10	0.05
Long-Distance Travel	0.25	0.15	0.30	0.15	0.15
School Pickup/Drop-off	0.20	0.15	0.10	0.30	0.25
Night-time Delivery	0.25	0.10	0.10	0.35	0.20

**Table 2:** Objective functions and their corresponding descriptions.

Objective Function	Description
$f_{\mathrm{length}}\left(p\right)$	Total Travel Distance
$f_{\mathrm{congestion}}\left(p\right)$	Cumulative Length of Congested Segments
$f_{\mathrm{time}}\left(p\right)$	Total Travel Time
$f_{\mathrm{risk}}\left(p\right)$	Cumulative Risk Value of the Route
$f_{\mathrm{avoidance}}\left(p\right)$	Degree of Route Penetration into Avoidance Zones

**Table 3:** Experimental design.

Experiment ID	Method Type	Description
Exp-1	Global A*	
Exp-2	Global DQN	
Exp-3	Global A* + local optimization via DQN	DQN performs local adjustment upon encountering an obstruction.

**Table 4:** Comparative analysis of experimental results.

Metric	Path Length (km)	Planning Time (s)	Risk Coefficient (0–1)	Dynamic Obstacle Avoidance Success Rate
A*	31.5 ± 0.2	0.3 ± 0.05	0.9 ± 0.05	10 ± 5%
DQN	38.2 ± 1.8	2.1 ± 0.3	0.3 ± 0.1	88 ± 4%
A*-DQN	32.8 ± 0.6	0.8 ± 0.1	0.4 ± 0.08	95 ± 3%

## Data Availability

The data presented in this study are available on request from the corresponding author due to confidentiality restrictions related to geographic information.
